# Spontaneous chylothorax identified during delivery

**DOI:** 10.1186/1749-8090-10-S1-A266

**Published:** 2015-12-16

**Authors:** Konstantinos Konstantinidis, Konstantinos M Soultanis, Antonia Gouma, Konstantinos Siafakas

**Affiliations:** 1 Department of Thoracic Surgery 251 Hellenic Airforce General Hospital, Athens, 11525, Greece; 2 Department of Thoracic Surgery, Sotiria Hospital, Athens, 11527, Greece

## Background/Introduction

Spontaneous (idiopathic) chylothorax during pregnancy is an extremely rare entity and symptoms can be attributed to other causes and lead to misdiagnosis. Management can be challenging.

## Aims/Objectives

To describe the mechanisms and management of gestational chylothorax

## Method

A 39 year old lady was referred to our department 15 days after delivery (caesarean section) of a healthy fetus due to left pleural effusion. She was complaining for shortness of breath and dry cough for the last 4 weeks of gestation and she was managed as a respiratory infection with no improvement to her symptoms. There was no attempt for a regular vaginal delivery (due to her request) and she was brought to the theatre for a Caesarian section. At the time of the procedure, diminished breath sounds were noticed on auscultation and after the delivery of a healthy fetus, a CXR followed by CT revealed a massive left pleural effusion. Diagnostic thoracentecis was performed and pleural fluid analysis showed chylothorax (milky fluid, triglyceride level: 340 mg/l).Despite the conservative management, chylothorax persisted so the patient was scheduled for VATS pleurodesis. A VATS chemical pleurodesis (left side) was performed under general anesthesia and single lung ventilation; no obvious leak was identified, talc was used and two chest drains were inserted. Twelve days after the procedure the triglyceride level of the pleural fluid was 45 mg/l and the output minimal, so diet was started gradually (100 cc of milk). The next day the triglyceride level was 160 mg/l and the total output 300 mls. Additionally, an ultrasound revealed thrombosis of the lower part of the left internal jugular and left innominate vein (previous central line) which obviously increased the chylothorax. On the day 25 a right posterolateral thoracotomy was performed and a mass ligation of the tissue between esophagus, azygous vein and the descending aorta was obtained. On the next two postoperative days the output from the chest drains was 780cc the 1st and 2380cc the 2nd day despite total parenteral nutrition. The patient was brought back to theatre, the incision reopened and oil was given through the nasogastric tube. A leak was noticed just next to the previous ligation stiches and the thoracic duct was dissected and ligated with 3-0 prolene pledgeted sutures. Introduction of oil didn't lead to leak this time.

## Results

Her postoperative period was complicated with septic episodes which were managed with appropriate changes of her antibiotic cover. Institution of low fat diet didn't result in new pleural effusion, the drains were removed and the patient was discharged on day 74.

## Discussion/Conclusion

Chylothorax following delivery has only been reported in individual cases and there are no large series describing the exact nature of the mechanism.

It is believed that high intraabdominal pressures generated by the Valsalva maneuver of the mother during pushing, or external pressure on the abdomen4 can result in high intrathoracic pressures which disrupt the thoracic duct into the pleural space. Idiopathic cases of gestational chylothorax have been reported and treated surgically after the delivery. In our patient, no Valsalva maneuver was performed and the patient admitted that her dyspnea and cough started the last month of gestation with no history of trauma or any change in her physical activity, which indicates the preexistence of the effusion. This lady has been followed up for 7 years after this episode and she is completely asymptomatic with no pleural effusions identified on x rays. Based on the experience gained from this case as well as from similar reports, early operative management of spontaneous chylothorax related to pregnancy is the most effective therapy.

## Consent

Written informed consent was obtained from the patient for publication of this abstract and any accompanying images. A copy of the written consent is available for review by the Editor of this journal.

